# Characterize the Complete Mitogenome of *Semiaquilegia guangxiensis* and Assess the Efficiency of the Mitochondrial Genes in Ranunculales Phylogeny

**DOI:** 10.1002/ece3.71165

**Published:** 2025-03-27

**Authors:** Zheng‐Juan Zhu, Xin‐Mei Qin, Peng‐Wei Li, Yong‐Bin Lu, Xiao‐Yuan Mo, Yuan Fang, Qiang Zhang

**Affiliations:** ^1^ College of Life Sciences Guangxi Normal University Guilin China; ^2^ Guangxi Key Laboratory of Plant Conservation and Restoration Ecology in Karst Terrain Guangxi Institute of Botany, Guangxi Zhuang Autonomous Region and Chinese Academy of Sciences Guilin China

**Keywords:** genomic reorganization, intergenomic DNA transfer, mitogenome, phylogenetics, *Semiaquilegia guangxiensis*

## Abstract

Plant mitochondrial genome (mitogenome) has crucial functions underpinning survive, development, and reproduction of organisms. However, the complete mitogenomes have been far less assembled and annotated than plastomes and even nuclear genomes in plants, due to their highly frequent and long repeat sequences and genomic structural variations. These further hinder the understanding of the mitogenome evolution and restrict potential applications in phylogenetic analyses. In this study, we sequenced, assembled, and annotated the complete mitogenome of *Semiaquilegia guangxiensis* and explored its evolution and usefulness in phylogenetics. The results showed that the mitogenome was composed of two independent molecules, which had a total length of 522,981 bp, a GC content of 45.69%, and 58 genes including 34 protein‐coding genes (PCGs), 21 tRNA genes, and three rRNA genes. A generalized codon usage preference was observed among the mitochondrial PCGs, and a total of 665 potential RNA editing sites were identified across the 34 mitochondrial PCGs, all of which were base C‐to‐U edits. Moreover, a large number of repetitive mitogenome sequences and chloroplast‐sourced sequences transferred to the mitogenome were detected. The largest collinear block identified between *S. guangxiensis* and *Paropyrum anemonoides* was 4282 bp in length. The phylogenetic analyses based on the mitochondrial gene sequences resolved the phylogenetic relationships within Ranunculales, in which *Semiaquilegia* was close to *Paropyrum*. The 19 PCGs were ranked according to their efficiencies on phylogenetic resolution based on several metrics, and the combined metric suggested *mat*R, *rps*3, and *nad*5 were the top three loci contributing most to phylogeny. As the first reported mitogenome in *Semiaquilegia*, our findings enrich the limited mitogenome library of plants, reecho the complex evolutionary dynamics of the mitogenome, and highlight the usefulness of mitochondrial gene sequences in phylogenetics.

## Graphical abstract

In this study, we assembled and annotated the whole mitogenome of *S. guangxiensis* and explored its evolution, including analyzing repeat sequences, codon usage, intergenomic transfer, and RNA editing sites. Moreover, we assessed and prioritized the usefulness of the mitochondrial genes in phylogenetics according to the combination of multiple metrics. Our findings reecho the complex evolutionary dynamics of the mitogenome and highlight the usefulness of mitochondrial gene sequences in phylogenetics.

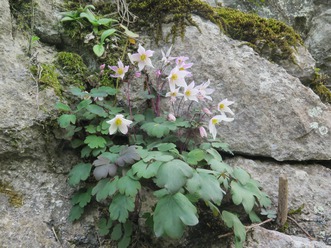

## 1 Introduction

As the vital energy factor within cells, mitochondria and their genomes play a crucial role in not only carrying genetic information but also actively participating in cellular metabolism and adaptive regulation processes (Cheng et al. 2021). The primary characteristics of plant mitogenome encompass rearrangement of gene order, profuse non‐coding sequences, abundance of repetitive sequences, a substantial amount of RNA editing, and immigration of sequences from plastid genomes (Cole et al. 2018; Li et al. 2024; Wang et al. 2024; Wu et al. 2022). These attributes contribute to the structural instability of plant mitogenome, which demonstrate large structural diversity encompassing unilinear, branched linear, cyclic, and combinations of cyclic and linear forms (Chen et al. 2024; Miao et al. 2024; Springer et al. 2019). These pose challenges in the assembly of plant mitogenome and only 673 complete plant mitogenomes were recorded in NCBI, far less than the plastid or even the nuclear genomes (Wang et al. 2024). Although highly variable in structure and arrangement of a mitogenome, the mitochondrial protein‐coding genes exhibit a relatively conserved nature, (some of) which have been tentatively used in phylogenetic studies (Li et al. 2023; Liu, Wu, et al. 2023; Qiu et al. 2010). However, the efficiency or resolution of the mitochondrial genes in phylogenetic inferences remains limited explored yet.

**FIGURE 1:**
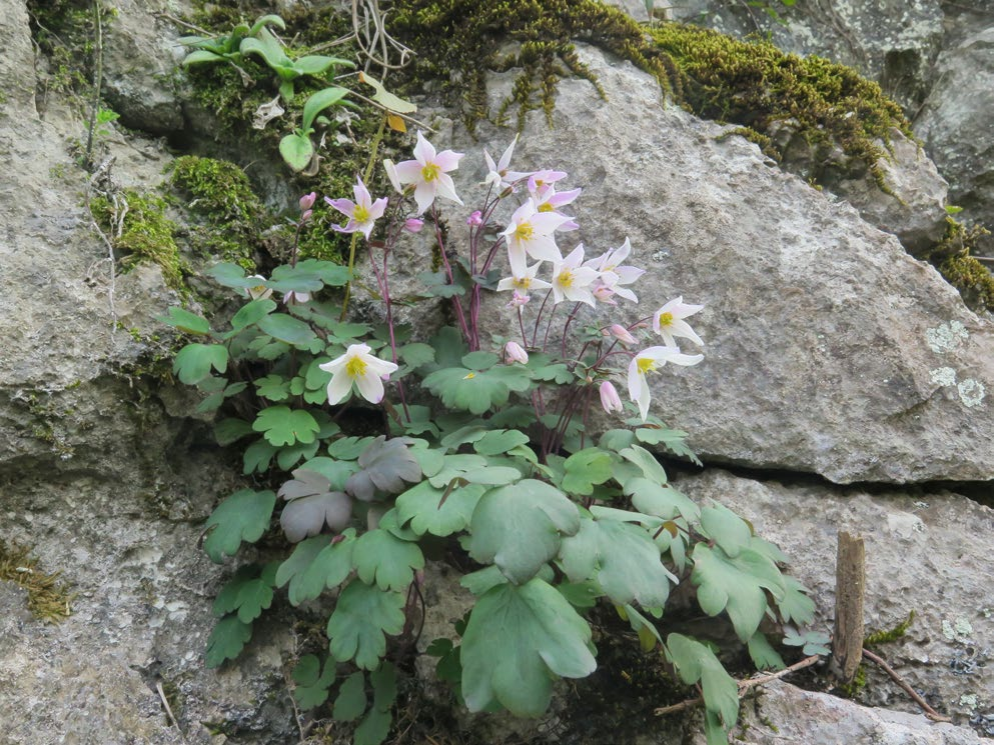
*Semiaquilegia guangxiensis* in the limestone habitat, North Guangxi, China. Photograph by Qiang Zhang.

The angiosperm family Ranunculaceae encompasses approximately 2500 known species, many of which have high medicinal and horticultural values (Yisilam et al. 2023). The family, along with those in the order Ranunculales, has a key phylogenetic position, which together form the basal most clade in eudicots. The family perhaps possesses the most diverse flowers and the widest distribution, and the most variable habitats or niches among all angiosperm families (Cheng et al. 2023; Emadzade and Hörandl 2011; Wang et al. 2016; Zhai et al. 2019). *Semiaquilegia* is a small genus in Ranunculaceae, which comprises four species that are mainly found in China, as well as Korea and Japan (Hsu et al. 2004; Huang et al. 2017; Son et al. 2017; Zhou et al. 2019). Notably, the four species have distinct habitats. *Semiaquilegia adoxoides*, a medicinal plant, has the widest distribution from South China to Korea and Japan and grows on soils as well as variable rock surfaces in open and shaded areas. The other three species each have extremely narrow distribution and are habitat specialists. Among them, *S. guangxiensis* is endemic to limestone habitat under forests in North Guangxi (Figure 1), South China, while *S. danxiashanensis* is only found in Danxia Mountain in Guangdong, South China, and *S. quelpaertensis* is only known from Korea (Huang et al. 2017; Son et al. 2017; Zhou et al. 2019). Previous phylogenetic analyses were based on plastid as well as nuclear loci, which indicated that *Semiaquilegia* formed a clade with *Urophysa* and *Aquilegia* in Ranunculaceae, but the relationships among the three genera remain uncertain (Wang et al. 2007; Yu et al. 2019; Zhai et al. 2019). In *Semiaquilegia*, the interspecific relationships were only investigated based on the plastid and nuclear ribosomal ITS sequences, which indicated *S. guangxiensis* diverged first from *S. adoxoides* and *S. danxiashanensis* (Qin et al. 2024; Zhou et al. 2019). So far, no mitogenome of *Semiaquilegia* has been assembled and used in phylogenetics, although 15 complete mitogenomes of other species in Ranunculales have been publicly available in NCBI.

In this study, we performed sequencing, assembly, and annotation of the mitogenome for *S. guangxiensis* . We then analyzed the mitogenome characteristics, including gene content, codon usage preference, repetitive sequence, RNA editing, and intergenomic gene transfer. Furthermore, we analyzed the phylogenetic relationships and collinearity with the published mitogenomes of Ranunculaceae. This study enriches the limited mitogenome library, reechoes the complex evolution of mitogenome, and highlights the usefulness of mitochondrial gene sequences in plant phylogenetics.

## 2 Materials and Methods

### 2.1 DNA Extraction and Sequencing

*Semiaquilegia guangxiensis* is not a protected species, and the plant individuals we collected in the wild are not owned by any department or person, so that need not any collection permission or license. To obtain the mitochondrial as well as plastid genome, fresh leaves were collected and promptly frozen in liquid nitrogen. Subsequently, total DNA was extracted using the CTAB method (Porebski et al. 1997), and its concentration and integrity were evaluated using the Quibit and NanoDrop instruments. High‐quality DNA samples with a predominant band size exceeding 30 kb were selected. The selected DNA was randomly fragmented into segments ranging from 15 to 18 kb using a covaris ultrasonic disruptor. Then, the DNA fragments were enriched and purified using magnetic beads. The fragmented DNA was subjected to damage repair and end repair procedures. Stem‐loop‐shaped sequencing adapters were attached to both ends of the DNA fragments, and any failed ligation products were eliminated using exonuclease treatment. The constructed library was sequenced by the PacBio Sequel IIe platform. Both the library construction and sequencing procedures were performed at Novegene Company. It finally generated a total of 28 Gb of sequence reads, with an average read length of 17,806 bp and an N50 of 18,492 bp.

### 2.2 Assembly and Annotation of Mitogenome

Firstly, mitochondrial genomic DNA sequence was assembled based on the PacBio HiFi long‐reads using the Flye software (v2.9.1‐b1780) (Kolmogorov et al. 2019) with the default parameters. The graphical assembly results in GFA format were obtained. All the assembled contigs in FASTA format were used to create a database using the makeblastdb. Subsequently, the BLASTN program (v2.13.0) (Chen et al. 2015) was employed with *Arabidopsis thaliana* and *Paropyrum anemonoides* mitogenomes as the query sequences, respectively, to identify contigs containing mitogenome sequences, using the parameters “‐evalue 1e‐5‐outfmt 6‐max_hsps 10‐word_size 7‐task blastn‐short.” Visualization of the GFA file was performed using Bandage software (v0.8.1) (Wick et al. 2015) and contigs containing mitochondrial sequences were selected based on the results of BLASTN to obtain a draft mitogenome map for *S. guangxiensis* .

The protein‐coding genes (PCGs) of the mitogenome were annotated using GeSeq (v2.03) (Tillich et al. 2017) with *P. anemonoides* (NC_072536), *A. thaliana* (NC_037304), and *Liriodendron tulipifera* (NC_021152) as the references. For tRNA annotation, tRNAscan‐SE (v2.0.11) (Lowe and Eddy 1997) was employed. Additionally, rRNA annotation for the mitogenome was carried out using BLASTN. Any annotation errors in the mitogenomes were manually curated and corrected using Apollo (v1.11.8) (Lewis et al. 2002).

### 2.3 Analysis of Codon Usage and Repeated Sequences

The PCGs of the mitogenome were extracted using Phylosuite (v1.1.16) (Zhang et al. 2020). Subsequently, the CAI module in Python was utilized to conduct codon usage bias analysis and calculate the relative synonymous codon usage (RSCU) values, and the results were visualized using ggplot2 (Wickham 2016). Simple sequence repeats (SSRs) were detected using Misa (v2.1) (https://webblast.ipk‐gatersleben.de/misa/) (Beier et al. 2017), establishing motif size thresholds at 10, 5, 4, 3, 3, and 3 for 1–6 nucleotides, respectively. Tandem repeats were identified using Tandem Repeats Finder (v4.09) (https://tandem.bu.edu/trf/trf.unix.help.html) (Benson 1999) with default parameters. Additionally, dispersed repeats were recognized by employing Reputer (https://bibiserv.cebitec.uni‐bielefeld.de/reputer/) (Kurtz et al. 2001), capturing forward (F), palindromic (P), reverse (R), and complementary (C) repeats. The results were visualized using ggplot2 (Wickham 2016).

### 2.4 Intergenomic Homology Analysis and Prediction of RNA Editing Events

The homologous sequences between the assembled mitochondrial and chloroplast genomes (MTPTs) were identified using BLASTN (v2.13.0) (Chen et al. 2015). Specific parameters, “‐evalue‐1e‐5, ‐word_size 10, ‐outfmt 6,” were set in this task. The visualization of the results was facilitated using Circos (v0.69.9) (Zhang et al. 2013). All the PCGs encoded by the mitogenome of *S. guangxiensis* were used as the input files to predict the RNA editing sites using Deepred‐Mt (Edera et al. 2021) based on the convolutional neural network (CNN) model. All results with a probability value greater than 0.9 were retained.

### 2.5 Collinearity and Phylogenetic Analyses

To investigate the collinearity, the mitogenome sequences of 10 species within Ranunculales were subjected to pairwise comparisons using the BLASTN (Chen et al. 2015), with the following parameters: ‐evalue 1e‐5, ‐word_size 7, and‐outfmt 6, and a minimum threshold of 80% identity and 500 base pairs in length for comparison results. Subsequently, the multiple synteny plot between *S. guangxiensis* species and the other species of Ranunculales was generated using the MCscanX software (Wang et al. 2012).

We downloaded the mitogenomes sequences of 17 species from NCBI, representing 15 species of Ranunculales, two species of Proteales. The two species from Proteales were designated as outgroup. Subsequently, we utilized PhyloSuite software (v1.1.16) (Zhang et al. 2020) to extract conserved genes, conducted multiple sequence alignment analysis using MAFFT software (v7.505) (Katoh and Standley 2013), and then employed IQ‐TREE software (v1.6.12) (Nguyen et al. 2015) to construct a phylogenetic tree based on the maximum likelihood method with parameters set as “‐alrt 1000‐B 1000.” The result of phylogenetic analysis was visualized using ITOL software (v6) (Letunic and Bork 2019).

Several metrics were used to measure the efficiency of the 19 PCGs on phylogenetic resolution. Sequence characters, including the number of variant sites (Vs) and the number of parsimony‐informative sites (Ps), were summarized using Mega software (v11.0) (Tamura et al. 2021). The average support values of the single‐gene trees were counted using python script. The Robinson‐Foulds (RF) and Quartet tree distances between each single‐gene tree and the reference concatenation‐based tree were calculated using the R packages phangorn and Quartet (Sand et al. 2014; Schliep 2011), respectively. Scores that is, rank orders, were given for each of the PCGs under each metric. Finally, each PCG was ranked according to the combined score of these individual metric scores.

## 3 Results

### 3.1 Structural Characteristics of the Mitogenome of *S. guangxiensis*

**FIGURE 2:**
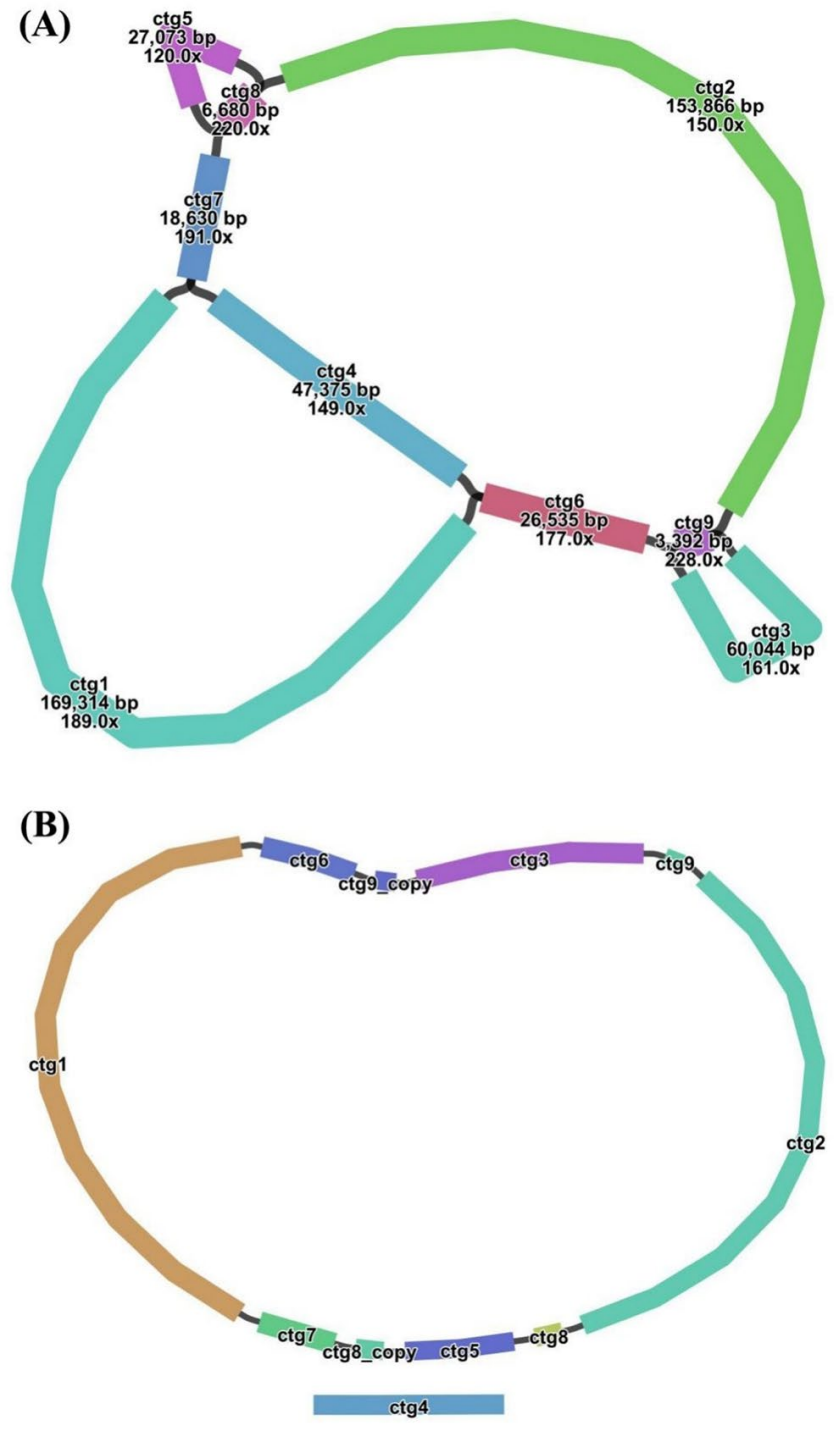
Assembly results of *Semiaquilegia guangxiensis* mitogenome. (A) Schematic of *S. guangxiensis* mitogenome assembly; (B) Main conformations of *S. guangxiensis* mitogenome. The specific solution paths are ctg1‐ctg7‐ctg8_copy‐ctg5‐ctg8‐ctg2‐ctg9‐ctg3‐ctg9_copy‐ctg6 (Circular) and ctg4 (Linear).

Using the two reference mitogenomes of *A. thaliana* and *P. anemonoides* yielded the same schematic of the mitogenome of *S. guangxiensis* . We employed the Bandage software to visualize the schematic of the mitogenome assembly, as depicted in Figure 2A. This schematic comprises nine nodes, with the corresponding length and sequencing depth indicated for each node. Each node represents an assembled contig. If two nodes are interconnected by a black line, it signifies the presence of overlapping regions between the two sequences (contigs). These sequences collectively form a complex multi‐branched closed genome structure. It was noted that the branched circular structure we presented was just one of multiple possible scenarios and was used here merely as an example. For several critical nodes with branching (ctg4, ctg8, ctg9), long‐reads were utilized to resolve the ambiguities. The long‐reads were mapped onto the related sequences at the branching nodes. The presence of a long‐read consecutively aligning with two connected sequences along the black line indicated that the long‐read supports the connection between the two sequences. In the cases where multiple alternative connections exist at a branching node, connections supported by a greater number of long‐reads were prioritized. The sequences of the circular contig (mitochondrial chromosome 1, MtChr 1) and the linear contig (MtChr 2) obtained after resolving the multifurcated nodes based on the long‐read data are shown in Figure 2B.

**FIGURE 3:**
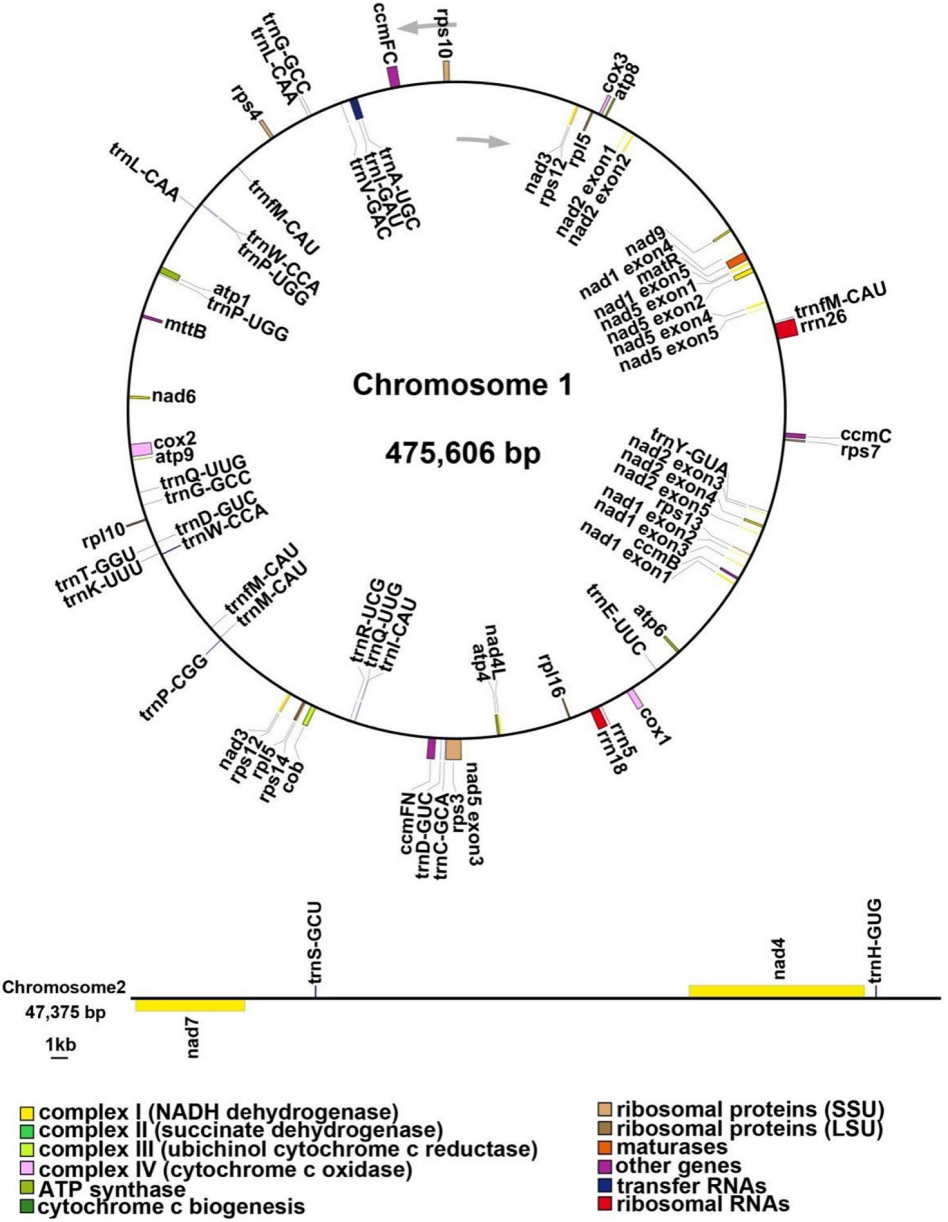
The mitogenome map of *Semiaquilegia guangxiensis* with annotation. The genes drawn inside and outside the circle are transcribed clockwise and counterclockwise, respectively.

The two chromosomes of the mitogenome had a total length of 522,981 bp with a GC content of 45.69%, in which MtChr 1 had a length of 475,606 bp with a GC content of 45.48% and MtChr 2 had a length of 47,375 bp with a GC content of 47.85% (Figure 3; Table 1). A total of 34 unique protein‐coding genes (PCGs) including 24 unique core mitochondrial genes and 10 variable genes, 21 tRNA genes (with seven tRNAs being multi‐copy), and three rRNA genes were annotated. The core genes included five ATP synthase genes (*atp*1, *atp*4, *atp*6, *atp*8, and *atp*9), nine NADH dehydrogenase genes (*nad*1, *nad*2, *nad*3, *nad*4, *nad*4L, *nad*5, *nad*6, *nad*7, and *nad*9), four cytochrome c biogenesis genes (*ccm*B, *ccm*C, *ccm*FC, and *ccm*FN), three cytochrome c oxidase genes (*cox*1, *cox*2, and *cox*3), one membrane transport protein gene (*mtt*B), one maturase gene (*mat*R), and one ubiquinol cytochrome c reductase gene (*cob*). The variable genes included three large ribosomal subunit genes (*rpl*5, *rpl*10, *rpl*16) and seven small ribosomal subunit genes (*rps*3, *rps*4, *rps*7, *rps*10, *rps*12, *rps*13, *rps*14) (Table 2).

**TABLE 1:** Basic information of *Semiaquilegia guangxiensis* mitogenome.

Contigs	Type	Length	GC content
Chromosome 1 & 2	Branched circular	522,981 bp	45.69%
Chromosome 1	circular	475,606 bp	45.48%
Chromosome 2	Linear	47,375 bp	47.85%

**TABLE 2:** The mitogenome encoding genes of *Semiaquilegia guangxiensis*.

Group of genes	Name of genes
ATP synthase	*atp*1, *atp*4, *atp*6, *atp*8, *atp*9
NADH dehydrogenase	*nad*1, *nad*2, *nad*3 (×2), *nad*4, *nad*4L，*nad*5, *nad*6, *nad*7, *nad*9
Cytochrome *b*	*cob*
Cytochrome *c* biogenesis	*ccm*B, *ccm*C, *ccm*FC, *ccm*FN
Cytochrome *c* oxidase	*cox*1, *cox*2, *cox*3
Maturases	*mat*R
Protein transport subunit	*mtt*B
Ribosomal protein large subunit	*rpl*5 (×2), *rpl*10, *rpl*16
Ribosomal protein small subunit	*rps*3, *rps*4, *rps*7, *rps*10, *rps*12 (×2), *rps*13, *rps*14
Ribosome RNA	*rrn*5, *rrn*18, *rrn*26
Transfer RNA	*trn*A‐UGC, *trn*C‐GCA, *trn*D‐GUC (×2), *trn*E‐UUC, *trn*fM‐CAU (×3), *trn*G‐GCC (×2), *trn*H‐GUG, *trn*I‐CAU, *trn*I‐GAU, *trn*K‐UUU, *trn*L‐CAA (×2), *trn*M‐CAU, *trn*P‐CGG, *trn*P‐UGG (×2), *trn*Q‐UUG (×2), *trn*R‐UCG, *trn*S‐GCU, *trn*T‐GCU, *trn*V‐GAC, *trn*W‐CCA (×2), *trn*Y‐GUA

*Note:* The numerical value enclosed in parentheses signifies the number of copies of the gene, such as (×2) representing the existence of two copies.

### 3.2 Codon Usage of PCGs

**FIGURE 4:**
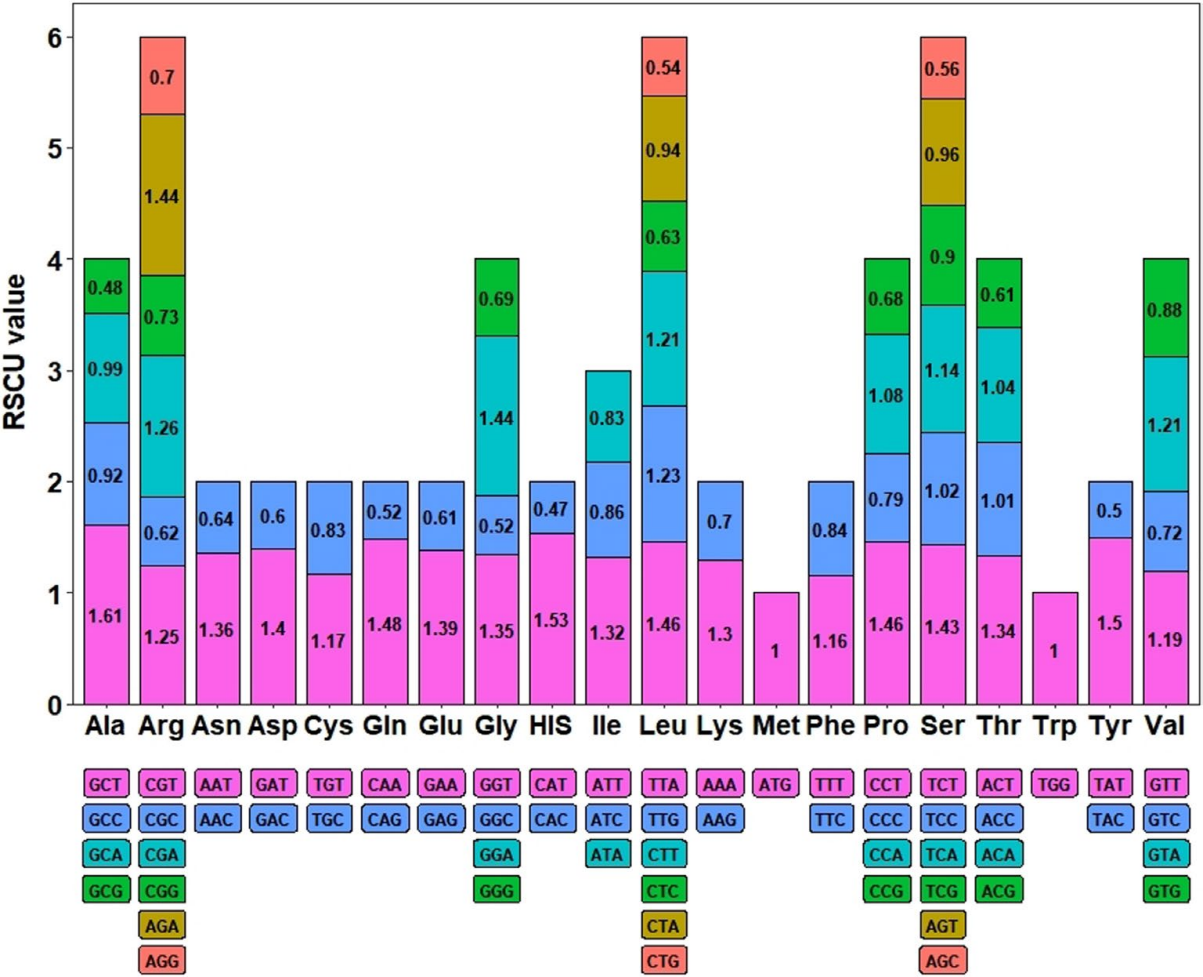
Codon usage of 20 amino acids in the mitogenome protein‐coding genes (PCGs) of *Semiaquilegia guangxiensis*. The codons are indicated by different colors in the histogram, and the numerical values on the histogram represent the relative synonymous codon usage (RSCU) of each amino acid for its codons.

The codon usage bias analysis was conducted for 34 unique PCGs of *S. guangxiensis* mitogenome. Codons with relative synonymous codon usage (RSCU) values greater than one are considered to be preferentially used for amino acids. The results showed (Figure 4) that, except for the start codon ATG and tryptophan (Trp, TGG), which both had RSCU values of one, we observed a generalized codon usage preference among the mitochondrial PCGs, with a total of 31 codons having an RSCU > 1. For example, alanine (Ala) had a high preference for GCT, with the highest RSCU value of 1.61, while histidine (His) had the lowest frequency of 0.47 of CAC use.

### 3.3 Analysis of Repeat Sequences

**FIGURE 5:**
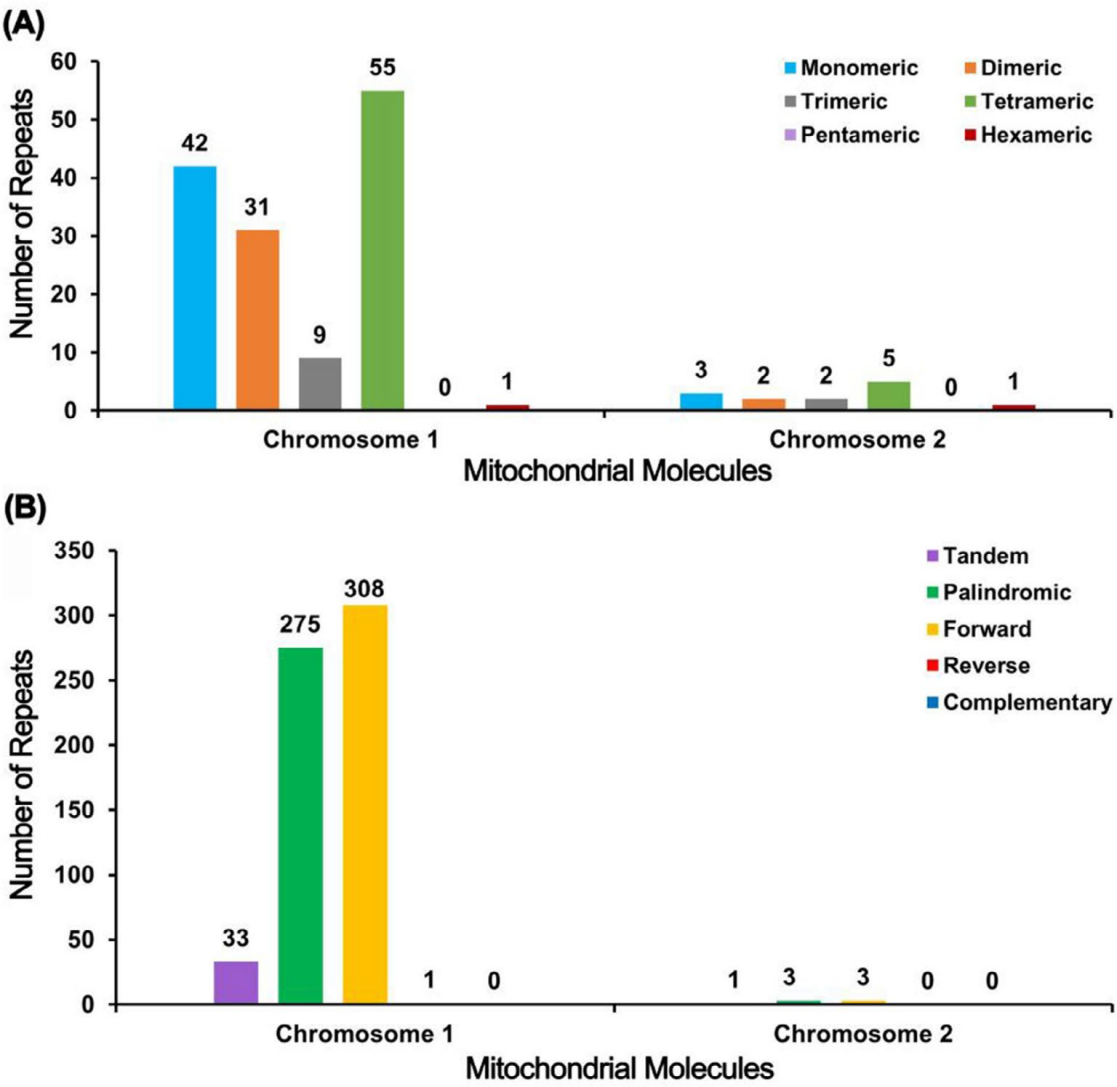
Histogram of repetitive sequence analyses. (A) The horizontal axis represents simple sequence repeat (SSR) types, while the vertical axis shows the number of repetitive fragments. (B) The horizontal axis shows the repeat types, while the vertical axis illustrates the number of repetitive fragments.

The repeat sequence analysis of MtChr 1 in the *S. guangxiensis* mitogenome identified a total of 138 SSRs, including 42 (30.43%) monomer SSRs, 31 (22.46%) dimer SSRs, nine (6.52%) trimer SSRs, 55 (39.86%) tetramer SSRs (the largest number), and one (0.72%) hexamer SSR. No pentamer SSRs were detected (Figure 5A; Table S1). Additionally, 33 tandem repeat sequences with a matching degree greater than 68% and a length ranging from 10 to 41 bp were identified in MtChr 1 (Figure 5B; Table S2). The dispersed repeat sequences in MtChr 1 were also examined, revealing 584 pairs of repeats with a length greater than or equal to 30 bp, among which 275 pairs were palindromic repeats, 308 pairs were forward repeats, and one pair was a reverse repeat. No complementary repeat was detected (Figure 5B; Table S3). The longest palindromic repeat was 17,762 bp, and the longest forward repeat was 6681 bp (Table S3).

In MtChr 2, 13 SSRs were found, including three (23.08%) monomer SSRs, two (15.38%) dimer SSRs, two (15.38%) trimer SSRs, five (38.46%) tetramer SSRs (the largest number), and one (7.69%) hexamer SSR. Similarly, no pentamer SSR was detected in MtChr 2 (Figure 5A; Table S4). In MtChr 2, there was one tandem repeat sequence with a matching degree of 100% and a length of 14 bp (Figure 5B; Table S5). The dispersed repeat sequences in MtChr 2 showed six pairs of repeats with a length greater than or equal to 30 bp, among which three pairs were palindromic repeats, three pairs were forward repeats, and no reverse or complementary repeat was detected (Figure 5B; Table S6). The longest palindromic repeat was 33 bp, and the longest forward repeat was 57 bp (Table S6).

### 3.4 Homology Analysis Between Organelle Genomes

**FIGURE 6:**
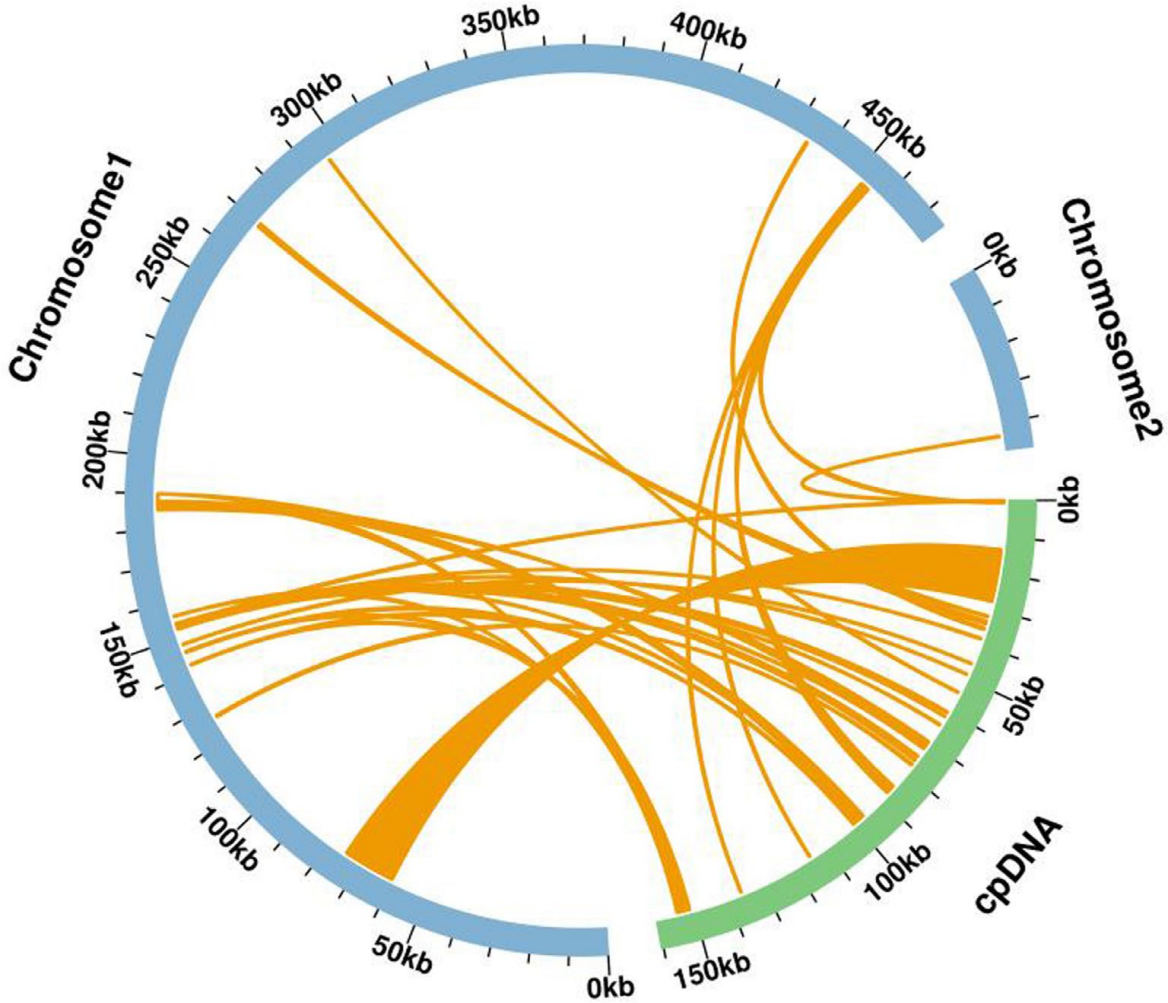
Homology analysis of the mitochondrial and chloroplast genomes of *Semiaquilegia guangxiensis*. The blue arcs represent the mitogenome, the green arc represents the chloroplast genome, and orange lines between the arcs correspond to homologous sequence fragments.

Based on sequence similarity analysis, a total of 30 homologous sequences were identified as mitochondrial plastid sequences (MTPTs) in *S. guangxiensis* , with a total length of 26,967 bp, accounting for 5.16% of the total mitogenome length (Figure 6). Among them, MTPT1 was the longest, with a length of 14,283 bp. Annotation of these homologous sequences revealed 19 complete genes on the 30 homologous sequences, including 13 PCGs (*atp*H, *atp*I, *rps*2, *rpo*C2, *rpo*C1, *rpo*B, *inf*A, *rps*8, *rpl*14, *pet*L, *pet*G, *psa*J, *rpl*33) and 6 tRNA genes (*trn*L‐CAA, *trn*H‐GUG, *trn*D‐GUC, *trn*W‐CCA, *trn*P‐UGG, *trn*M‐CAU). Additionally, some incomplete genes from chloroplasts were also observed on these homologous sequences, such as *ycf*2, *rpl*16, *rpl*33, *psb*B, *psb*D, and others (Table S7).

### 3.5 RNA Editing Site Prediction

**FIGURE 7:**
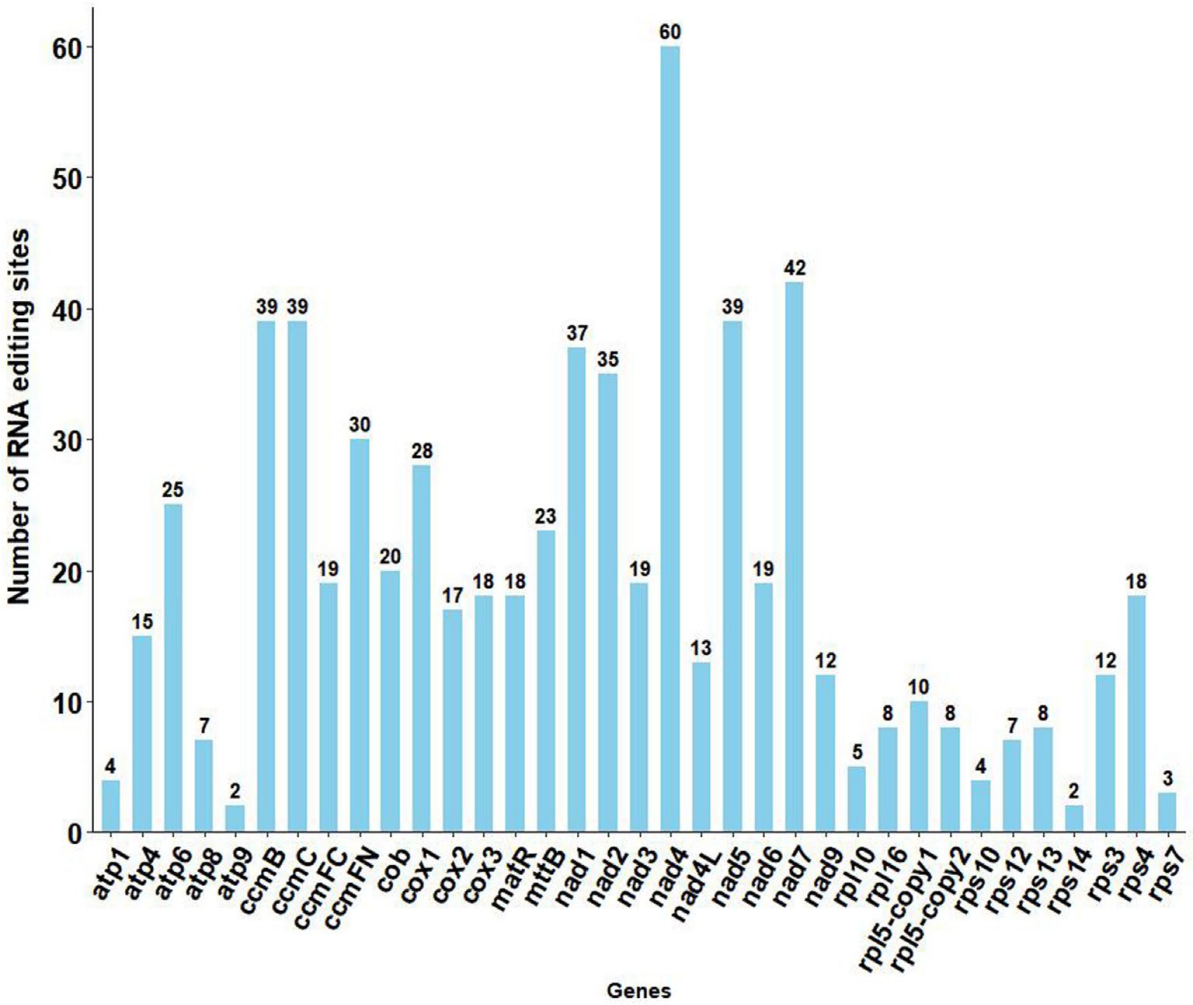
Number of RNA editing sites predicted for the 34 protein‐coding genes (PCGs) in the *Semiaquilegia guangxiensis* mitogenome.

RNA editing events were identified in 34 PCGs of *S. guangxiensis* mitogenome by employing a Convolutional Neural Network (CNN) model. With a predictive performance of 0.9 (where 0 indicated no editing and 1 indicated editing, with a higher value approaching 1 suggesting a higher probability of editing), a total of 665 potential RNA editing sites were identified across the 34 mitochondrial PCGs, all of which were base C‐to‐U edits. Furthermore, the majority of these RNA editing sites predominantly occurred at the first or second position (Table S8). Among the mitochondrial genes, the *nad*4 gene exhibited the highest number of RNA editing sites, with 60 identified, and it was followed by the *nad*7 gene, which underwent 42 RNA editing events. Both the *atp*9 and *rps*14 genes had the least (only two) editing sites (Figure 7). A total of 46 amino acid variations were associated with RNA editing, among which 45.71% of the amino acids showed no change in hydrophobicity, 45.11% of the amino acids were predicted to undergo a transition from hydrophilic to hydrophobic, and 8.87% were predicted to shift from hydrophobic to hydrophilic (Table S9). Additionally, RNA editing events that could potentially introduce stop codons were discovered in the *ccm*FC and *rps*10 genes. Our results also showed that the amino acids predicted for the edited codons showed a leucine bias, which was supported by the fact that 43.60% (290 sites) of the edits were converted to leucine.

### 3.6 Collinearity Analysis

**FIGURE 8:**
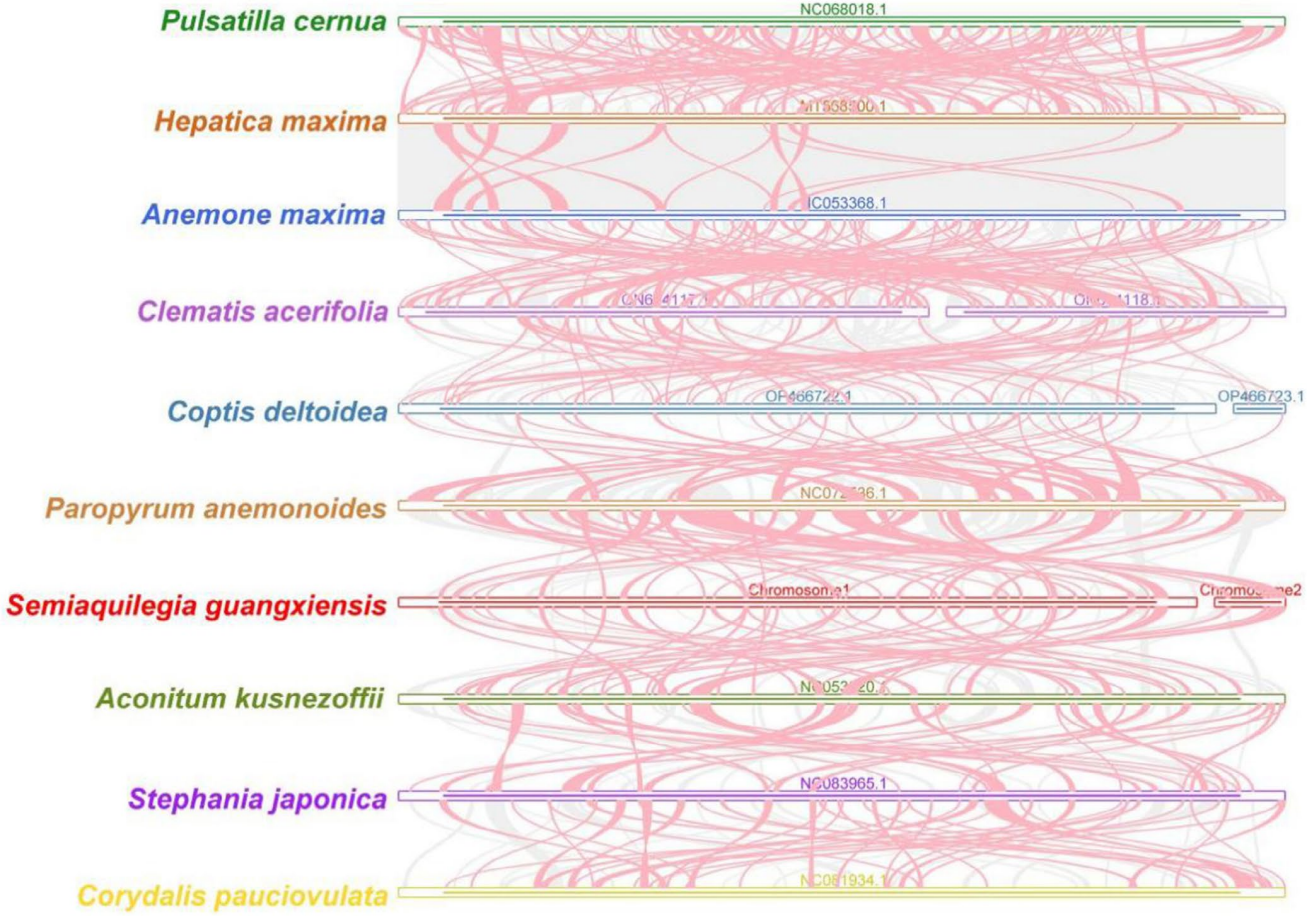
Collinearity analysis of *Semiaquilegia guangxiensis* and the closely related species in Ranunculales. The red lines indicate where inversions have occurred, while the gray areas indicate syntenic regions of high homology.

In the collinearity analysis, collinear blocks shorter than 0.5 kb were excluded. A large number of homologous collinear blocks were detected between *S. guangxiensis* and the closely related species in the Ranunculales, but these collinear blocks were relatively short. When compared with the sampled closest relative *P. anemonoides*, the largest collinear block was only 4282 bp ( Table S10). Furthermore, some blank regions were identified, which denoted unique sequences in *S. guangxiensis* and did not share homology with the other species (Figure 8). The results indicated that the collinear block arrangements among the mitogenomes of these 10 species were inconsistent, and the mitogenome of *S. guangxiensis* had undergone genomic rearrangements compared with the closely related species. However, there were many relatively short homologous sequences between the species, especially gene fragments, indicating that genes were conserved across the mitogenomes (Figure 8).

### 3.7 Phylogenetic Analyses

**FIGURE 9:**
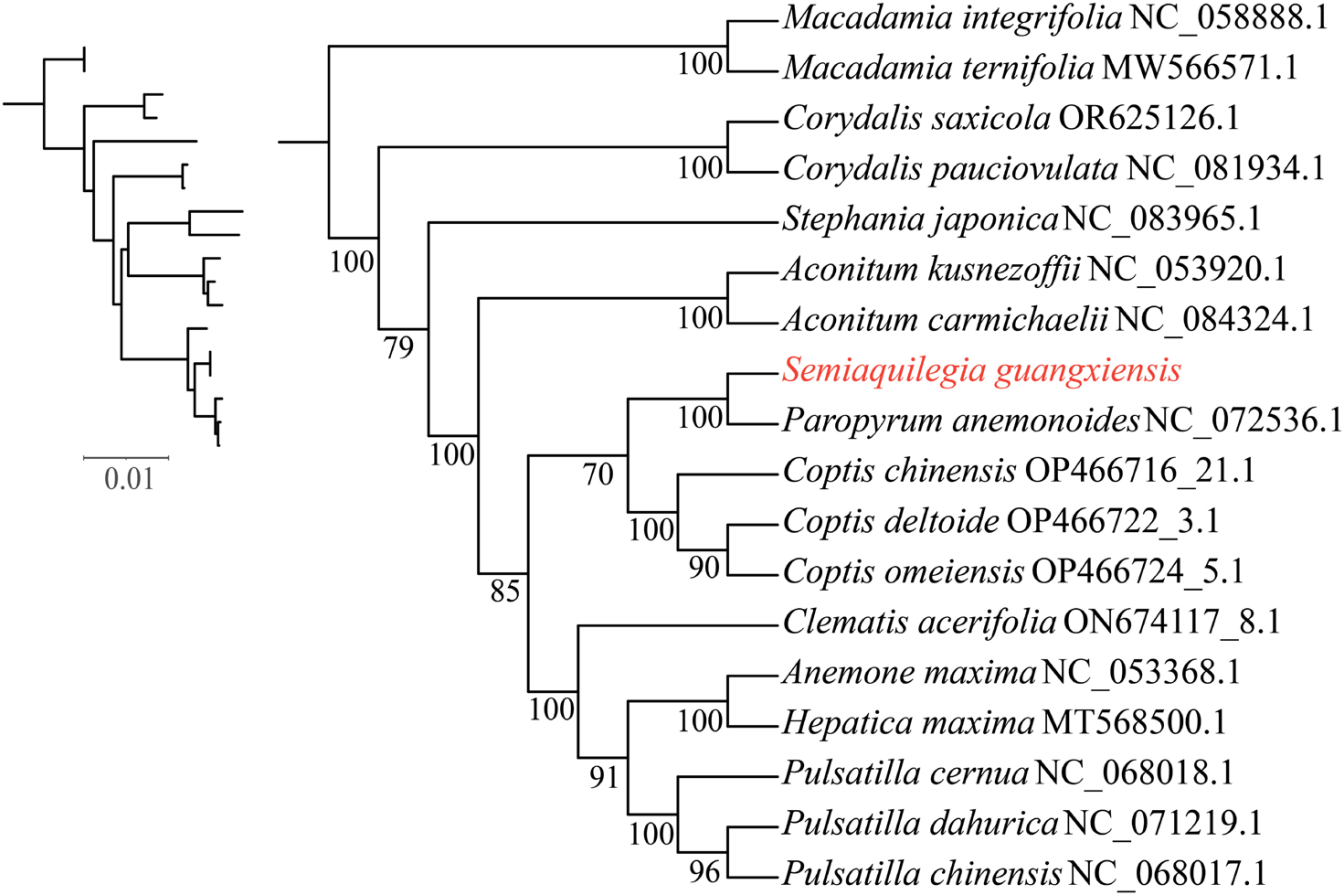
A maximum likelihood tree based on the nucleotide sequences of 19 conserved core mitochondrial protein‐coding genes (PCGs). *Macadamia integrifolia* and *M. ternifolia* were chosen as the outgroup. The number at each node represents the bootstrap support. The Latin name of *Semiaquilegia guangxiensis* whose mitochondrial and plastid genome sequences were newly sequenced and assembled in this study is highlighted in red.

To explore the usefulness of mitochondrial genes in phylogenetics and the phylogenetic placement of *S. guangxiensis* in Ranunculales, a phylogenetic tree was constructed based on the DNA sequences of 19 conserved mitochondrial protein‐coding genes (*atp*1, *atp*4, *atp*8, *atp*9, *ccm*B, *ccm*C, *ccm*FN, *cob, cox*3, *mat*R, *nad*1, *nad*2, *nad*3, *nad*4L, *nad*5, *nad*6, *nad*7, *rps*3, *rps*12). The results revealed that 13 species from the Ranunculaceae clustered into one clade (BS = 100), and *S. guangxiensis* was the closest to *P. anemonoides* in Ranunculaceae, with the highest bootstrap support (BS =100). In addition, *Coptis* was the sister of both *Semiaquilegia* and *Paropyrum* (BS = 70); *Clematis* was suggested to be the sister of the clade (BS = 91) comprising the three taxa, in which *Pulsatilla* was the sister of both *Hepatica* and *Anemone* (BS = 100) (Figure 9). Furthermore, the five metrics, that is, number of variable sites, number of parsimony‐informative sites, Robinson‐Foulds distance, Quartet distance, and average bootstrap support, showed basically consistent rank orders of the PCGs. The combined metric manifested that *mat*R, *rps*3, and *nad*5 had the best scores and were the top three markers that could be the most useful for phylogenetic inferences (Table 3).

**TABLE 3:** Values of the phylogeny‐related metrics and ranked orders in parentheses of the 19 protein‐coding genes (PCGs).

Name of genes	Number of variable sites	Number of parsimony‐informative sites	Average bootstrap support (%)	Robinson‐Foulds distance	Quartet distance	Rank
*mat*R	447 (2)	259 (2)	87.44 (1)	8 (1)	3338 (2)	1
*rps*3	718 (1)	437 (1)	82.82 (2)	10 (2)	3444 (3)	2
*nad*5	261 (5)	180 (5)	80.00 (3)	18 (5)	5142 (5)	3
*ccm*FN	311 (4)	200 (4)	73.32 (6)	24 (7)	5062 (4)	4
*atp*1	320 (3)	251 (3)	78.39 (5)	22 (6)	7584 (11)	5
*atp*8	145 (9)	119 (7)	79.56 (4)	18 (5)	5666 (8)	6
*atp*4	141 (11)	96 (11)	73.03 (7)	14 (3)	2426 (1)	7
*nad*2	207 (6)	136 (6)	72.68 (8)	24 (7)	8646 (12)	8
*ccm*C	154 (8)	110 (9)	64.50 (11)	24 (7)	6246 (9)	9
*nad*7	142 (10)	108 (10)	70.15 (9)	24 (7)	6644 (10)	10
*cox*3	123 (12)	87 (12)	63.50 (12)	16 (4)	5438 (6)	11
*cob*	180 (7)	115 (8)	67.29 (10)	34 (10)	8673 (13)	12
*nad*1	89 (14)	58 (15)	53.47 (15)	22 (6)	5638 (7)	13
*ccm*B	101 (13)	71 (13)	60.89 (13)	28 (8)	8794 (14)	14
*nad*6	89 (14)	67 (14)	57.21 (14)	32 (9)	10,832 (15)	15
*atp*9	71 (16)	55 (16)	50.35 (16)	44 (13)	16,917 (16)	16
*nad*3	54 (17)	42 (17)	49.53 (18)	38 (11)	20,653 (18)	17
*nad*4L	47 (18)	35 (19)	50.21 (17)	40 (12)	19,648 (17)	18
*rps*12	82 (15)	38 (18)	36.15 (19)	48 (14)	25,778 (19)	19

## 4 Discussion

### 4.1 Characteristics of the Mitogenome of *S. guangxiensis*

So far, only the mitogenome of *P. anemonoides* has been reported in the tribe Isopyreae of Ranunculaceae (Yisilam et al. 2023), and the mitogenome of *S. guangxiensis* assembled in this study is the first reported complete mitogenome in the genus *Semiaquilegia*. Previous studies have predominantly characterized mitogenomes as circular in structure. For instance, the mitogenomes of various species, including *Pereskia aculeata* , *Genlisea tuberosa*, *Camellia sinensis*, and *Fagopyrum esculentum*, each have been found to contain single or multiple circular DNAs (Logacheva et al. 2020; Matos et al. 2022; Zhang et al. 2019, 2023). Moreover, it is important to note that the plant mitogenome should be viewed as a complex and dynamically assembled collection of smaller circular and linear DNA molecules rather than simply linear DNA collections (Anderson et al. 2021; He et al. 2021; Li et al. 2022). Unlike the previous studies, particularly those on Ranunculales (Szandar et al. 2022; Yisilam et al. 2023), the mitochondrial DNA structure of *S. guangxiensis* consists of a loop and a linear structure. The results of the present study support the idea that multiple structures exist for the plant mitogenome.

Although the size of angiosperm mitogenome varies considerably between or even within species, the number of functional genes encoded is relatively conserved (Clifton et al. 2004; Kubo and Newton 2008; Ogihara et al. 2005; Song et al. 2023). The mitogenome of *S. guangxiensis* contains 58 genes, generally in agreement with the types of genes encoded by the mitogenomes of other plants. These include genes responsible for encoding respiratory chain complexes I, II, III, IV, and V, multiple cytochrome *c* synthase subunits, and transfer RNAs (tRNAs) and ribosomal RNAs (rRNAs) (Kubo et al. 2007; Song et al. 2023; Sun et al. 2022). Some mitochondrial genes were frequently lost, including the genes encoding ribosomal proteins (*rpl* and *rps* genes) and the *sdh* gene (succinate dehydrogenase) (Adams et al. 2002; Bi et al. 2022). In *S. guangxiensis* , both *sdh*3 and *sdh*4 genes have been found to be absent. Furthermore, the loss of the *sdh*3 gene has been observed in *P. anemonoides* as well, which could have resulted from the same event that happened in the common ancestor of *P. anemonoides* and *S. guangxiensis* . There are a number of potential reasons for the loss of genes in mitochondria. One possibility is that the species no longer requires the function of the lost gene and therefore lost it by drift. Alternatively, the gene might have been transferred to the nuclear genome or its function might have been undertaken by other genes (Adams et al. 2001). Further investigation is required to determine the processes and precise underlying reasons for the mitochondrial gene loss.

### 4.2 Plastid‐Derived Sequences in the *S. guangxiensis* Mitogenome

Transfer of chloroplast sequence to mitogenome is common (Gui et al. 2016; Liu, Yuan, et al. 2023; Nguyen et al. 2020; Niu et al. 2024). Consistent with the findings in other species in Ranunculaceae (Yisilam et al. 2023), chloroplast sequences were inserted into different regions of the mitogenome in *S. guangxiensis* . The transfer could contribute to rearrangement of the mitogenome and promote genetic diversity (Shidhi et al. 2021). Similar to the amount/ratio (3%–11.5%) of MTPT in most other angiosperms (Alverson et al. 2010; Yurina and Odintsova 2016), the lengths of the transfer fragments in the *S. guangxiensis* mitogenome ranged from 30 bp to 14,284 bp, with the total sequence length accounting for approximately 4.71% of the total mitogenome length, suggesting that the enlargement of the mitogenome is partly ascribed to the integration of plastid‐derived sequences. Interestingly, the MTPT fragments contain multiple PCGs and tRNA genes maintaining intact, suggesting that they may still be functional. Even the incompletely transferred PCGs with premature stop codons cannot be directly excluded from being functional. In sum, the processes and functions of the migrant sequences remain unclear and require further study.

### 4.3 Usefulness of the Mitochondrial Genes in Phylogenetics

Nuclear and plastid sequences have been widely used for plant phylogenetic analyses (Mu et al. 2020; Wang et al. 2023), but mitochondrial sequences have been much more rarely used (Li et al. 2023; Liu Qian et al. 2023; Qiu et al. 2010). In this study, we analyzed the phylogenetic relationships of *Semiaquilegia* and other genera in Ranunculales based on 19 conserved mitochondrial protein‐coding genes. These results underscore the great potential of the mitochondrial genes for phylogenetic analyses within orders, despite the mitogenomes having been largely rearranged and only short collinear blocks being identified even between the sampled closest relatives *S. guangxiensis* and *P. anemonoides*. In addition, some controversial relationships with relatively low support based on plastid and nuclear sequences are resolved with higher support values by using mitochondrial genes in this study. For example, *Heptica* was more closely related to *Clematis* (BS = 23) than to *Anemone* in the study of Zhai et al. (2019) based on plastomic data, while it was the closest to a clade (BS = 83) consisting of three taxa, in which *Clematis* was sister to both *Anemone* and *Pulsatilla* (BS = 100) in the study of Wang et al. (2009) based on several plastid and nuclear ribosomal DNA sequences (Wang et al. 2009). However, in this study based on mitochondrial genes, *Clematis* was suggested to be the sister of the clade (BS = 91) comprising the three taxa, in which *Pulsatilla* was the sister of both *Hepatica* and *Anemone* (BS = 100). These relationships were more consistent with those based on the nuclear genes in the study of He et al. (2022).

Mitochondrial genes usually have slower mutation rates than plastid and nuclear genes, and thus may be better suited for reconstructing deep phylogenetic relationships (Qiu et al. 2010; Wei et al. 2022). The clearly‐resolved intergeneric relationships in Ranunculaceae/Ranunculales suggest that the mitochondrial genes also have potentials to resolve the phylogenetic relationships of relatively recently diverged taxa, although they are usually slowly‐evolving. In conclusion, the above findings suggest that mitochondrial genes provide complementary rather than redundant insights into the current phylogenetic relationships inferred from plastid and nuclear data. Furthermore, the rank order of suitability for phylogeny proposed in this study provides a preliminary reference for which mitochondrial genes should be isolated for phylogenetic analyses in priority.

## 5 Conclusion

In this study, we successfully assembled and annotated the whole mitogenome of *S. guangxiensis* with a total length of 522,981 bp and a GC content of 45.69%. A total of 58 genes were annotated, including 34 PCGs, 21 tRNA genes, and three rRNA genes. The codon preferences, repetitive sequences, genome homology, RNA editing events, phylogenetic relationships, and usefulness of individual mitochondrial genes in phylogenetics have been comprehensively analyzed. Our findings enrich the limited mitogenome library of plants, reecho the complex evolutionary dynamics of mitogenome, and highlight the usefulness of mitochondrial gene sequences in phylogenetics.

## Author Contributions

**Zheng‐Juan Zhu:** data curation (lead), formal analysis (lead), investigation (lead), methodology (lead), project administration (lead), validation (lead), visualization (lead), writing – original draft (lead), writing – review and editing (equal). **Xin‐Mei Qin:** conceptualization (lead), data curation (lead), formal analysis (lead), funding acquisition (equal), investigation (lead), methodology (lead), project administration (lead), validation (lead), visualization (lead), writing – original draft (lead), writing – review and editing (equal). **Peng‐Wei Li:** writing – review and editing (supporting). **Yong‐Bin Lu:** writing – review and editing (supporting). **Xiao‐Yuan Mo:** formal analysis (supporting), validation (supporting), visualization (supporting), writing – review and editing (supporting). **Yuan Fang:** formal analysis (supporting), validation (supporting), visualization (supporting), writing – review and editing (supporting). **Qiang Zhang:** conceptualization (lead), funding acquisition (equal), resources (lead), supervision (lead), writing – review and editing (lead).

## Conflicts of Interest

The authors declare no conflicts of interest.

## Supporting information


**Tables S1‐S10**.

## Data Availability

The complete mitochondrial and plastid genome sequences of *Semiaquilegia guangxiensis* are available at GenBank with accession numbers: PQ572709 (Chromosome 1), PQ572710 (Chromosome 2) and PQ584724. All the other mitogenome sequences used in this study are available in the NCBI Genome Database (https://www.ncbi.nlm.nih.gov/) under the GenBank accessions are listed after the Latin names of the species in the phylogenetic tree in Figure 9.
